# Observations on the Epidemic Yellow Fever of Natchez, and of the South-west

**Published:** 1841-11

**Authors:** John W. Monette

**Affiliations:** Washington, Mississippi


### WESTERN JOURNAL

OF

### MEDICINE AND SURGERY.

NOVEMBER, 1841.

Art. I.—*Observations on the epidemic Yellow Fever of Natchez, and of the South-west.* By John W. Monette, M. D. &c., of Washington, Mississippi.

In presenting to the medical profession, and to the public generally, the result of my observations and reflections, for nearly twenty years, on the subject of Yellow Fever as it occurs in the south-western portion of the United States, I deem it necessary and proper to premise as follows, viz: that the writer is a graduate of the Medical School of Transylvania in its most flourishing era; when the leading chairs were filled by those eminent men, Professors Dudley, Drake, and Caldwell. As a matter in course, the doctrines taught by them relative to Yellow Fever, were the rule of my faith; and such as are still entertained by most of the profession, in the middle and south-western portions of the United States. With implicit confidence in their information, judgment, and experience, I adhered to what I believed the true doctrine of Yellow Fever; a doctrine too, which none dare doubt, or attempt to controvert, without risk to his reputation as a man of science and deep medical learning.

By most of the Faculties in our northern and western schools, if we mistake not, it is taught as a settled doctrine, a "*res adjudicata,*" that Yellow Fever is a *high grade of Bilious Fever;* that it is produced locally by local causes, such as a peculiar miasm, or exhalation from certain putrescent matters; and especially from such as constitute ordinary city filth, in alleys, sewers, cellars, and more especially about wharves in commercial cities: that this fever thus generated is confined to the city or district where it originates: that it possesses no communicable properties: or that no exhalation or effusion is disengaged from the body laboring under its influence, which in any manner tends to reproduce that disease in others who are exposed to its influence: that there is no danger of this disease being transported from an infected district or city to one which is healthy; consequently that all quarantine restrictions upon the intercourse and commerce with infected cities and districts, are not only useless, oppressive, inefficient in excluding the disease, but are not sustained by the experience of the enlightened portion of the profession, as consistent with the present state of medical science, in the United States and Europe.

Such were the opinions of the writer; in the comfortable enjoyment of which, no doubt, he would have remained to this day, had not his lot been cast in a region, where it has been his business, from his earliest professional avocations, to see, examine, and treat this disease, under circumstances which compelled him to trace the origin, and causes, and all the circumstances under which it has visited some of the most healthy portions of the United States. Facts have been repeatedly presented which produce the irresistible conviction, that this disease, in the South, may be transported from one place to another, and there disseminated among a healthy population, so as to produce an epidemic. The circumstances requisite for the certain and speedy dissemination of this disease, are certainly more frequently combined in this latitude than in our northern and middle States. Facts, as observed at Natchez and other southern towns, are such as to confirm, in many instances, the doctrine taught and believed in southern Europe, as well as in some cities of the United States, and especially in those liable to frequent visitations of Yellow Fever.

The first epidemic Yellow Fever which came under our notice, was in 1823. Since that time, circumstances presented by every visitation, have gradually confirmed us in the convictions partially made at that time. Additional facts, and additional reasons have more fully convinced me, that the disease is generally, if not always, imported into Natchez as well as New Orleans, chiefly from Havana and the West Indies. In this belief, for twelve or fifteen years, the writer was almost the only one of the profession in the southwest, who had the courage to declare his sentiments publicly in favor of *importation.* Many who were present at every epidemic, like the great mass of mankind, looked upon the operations of nature, without inquiry, without observation, and without any close analysis of judgment; satisfied to follow blindly in the road laid down by visionary theorists; and which had been generally adopted as *orthodox* and *fashionable.* Ever since the epidemic of 1823, the writer has continued to urge these opinions upon the citizens of Natchez, as well as those of New Orleans; although opposed by the opinions of many excellent physicians, and by the ridicule of those who had nothing better to offer.

Now I have the satisfaction to find that my opinions on this subject are fully sustained, not only by the great body of observing and intelligent citizens, but also by some of the most eminent physicians of this state. Among these are my friends, Dr. McPheeters, and Dr. Davis, the present health-officer of

Natchez. The same opinions are likewise obtaining rapidly in New Orleans, Mobile, and Charleston, S. C. The day is not very remote, when New Orleans and other southern ports may be rendered as exempt from Yellow Fever, as Philadelphia and New York now are. A judicious, timely, and strict quarantine regulation, at each of our southern commercial cities and towns, by excluding this pestilence, will be the wise means of preserving annually hundreds and thousands of lives, and of extending the commerce and prosperity of the people. To the writer, this result would far outweigh all the approbation attainable, by adhering to established opinions, and fashionable errors. We of course expect to encounter the dissent and disapprobation of many distinguished medical men, who have, however, never enjoyed proper opportunities to acquire correct views of this disease in this latitude. I am sustained by many able and learned physicians of the south, when I declare, that our brethren in the great Ohio Valley are greatly mistaken, when they suppose they have seen *true Yellow Fever* north of the mouth of Ohio, unless when rapidly transported in steamboats. But to our subject.

Notwithstanding the great diversity of opinions entertained by medical men, relative to the origin and nature of Yellow Fever miasm, there are a few important facts generally admitted; and from these important deductions are drawn.

It is admitted on all sides that Yellow Fever is the product or effect of a certain deleterious miasm, or poisonous effluvium, operating upon a healthy system unacclimated to its action; that this miasm, malaria, or infection, wherever existing and diffused in the local atmosphere, operates its deleterious influences upon the system, through the medium of the lungs, or by being respired. It is admitted that when this malaria, infection, or miasm, is produced, or exists in any part of a city, in a steady high temperature of autumn, and among a crowded population, it will gradually diffuse itself into the surrounding air, which was previously healthy, and there produce Yellow Fever equally malignant, with that produced by it in the place of its origin. It is also admitted and well known, that when it diffuses itself through any part of a city, that the whole atmosphere to that extent becomes equally deleterious; and that this deleterious property diffuses itself into houses, rooms, cellars, and avenues, and indeed into every recess, or interstice into which common air can penetrate: that it insinuates itself into the texture of porous articles, in which air largely enters: that ships in port, where Yellow Fever is epidemic, become thoroughly infected, and resist every disinfecting agent except a low temperature, which entirely neutralizes it: that persons from northern climates are peculiarly obnoxious to the influence of this miasm: and that strangers or unacclimated persons passing into an infected district, house, or ship, and there breathing that atmosphere for one or two minutes, or even less, will contract genuine Yellow Fever, and probably die with black vomit. It is also admitted and known, that any infected air, either in open space, in houses, ships, or any other place, becomes entirely deprived of its deleterious and morbific properties, so soon as the temperature is reduced to 32° of Fahrenheit: it is also well known that when a city or any portion is strongly infected, a storm of wind and rain may pass over the city, and even frost may neutralize or destroy all the external infection, while that in closed houses, ship-holds, and other close places, is unchanged until the temperature is reduced to about 32° of Fahrenheit. It must be admitted, that the *removal* of a house or a ship, or any other matter enclosing infected air, does not destroy the infectious virulence, while a uniform temperature is preserved. If this be admitted, it follows of course, that a ship, infected in one port, may sail to another port, and there produce Yellow Fever in those who breathe its contaminated atmosphere. The infected atmosphere of one ship, and especially where several infected ships are contiguous, will and does diffuse itself through the surrounding atmosphere; thus extending the sphere of its morbific influence, in the same manner, as it is admitted that an infected district in a city will finally infect the whole city, where communication is uninterrupted. Besides this process of extension, or of assimilating the surrounding air to its own infectious nature, hundreds of persons, from remote and opposite parts of a city, within a week or ten days, during the time an infected vessel lies in a healthy port, will have direct intercourse with the ship, and thus contract the disease, in the same manner in which persons from the country would do, by visiting an infected city or town. These persons subsequently are attacked with the disease fully developed, in opposite and remote parts of a city or town, where their dwellings happen to stand. In this manner, Yellow Fever, introduced by vessels or steamboats from the wharves, will show itself simultaneously about the wharves and in various remote quarters of the city. Thus those who look for a palpable and visible extension of the disease, like the spread of fire in a city, are at a loss to trace all the cases to one common source.

Again; we know that this *infected air*, confined in a house during the hot sultry weather of August and September, in the latitude of Natchez, will become more virulent than when first confined in that house. This has been fully exemplified during every visitation of Yellow Fever in Natchez. Houses, which were deserted and closed at the beginning of the epidemic, and when the general air was but slightly infected, and when all the inmates of that house were healthy, and continued so in their retreat, have become so strongly infected by being closed, that on being opened, after frost, and after all general appearance of the disease had subsided, they have been fatal to those who have entered, until thoroughly purified by ventilation and frost.

Again; it is well known that during an epidemic visitation at Natchez, equinoctial storms and rains have so swept out the free infection from the streets and open grounds, that the disease for a time appeared to have subsided: but the virulent infection contained in the houses, and which was beyond the influence of the storm and rain, required only a few warm calm days to disseminate itself as widely as ever.

Yellow Fever is indigenous to the tropics, and will not manifest itself epidemically much, if any, beyond the tropics, unless the infection is introduced from tropical ports in the West Indies. In these islands and other tropical ports of the Gulf of Mexico, Yellow Fever is indigenous, and exists among northern strangers, more or less during the whole year. In these ports it is confined exclusively to the strangers and unacclimated, while those who have been acclimated, or who are natives, are exempt from its influence. Yet as each additional case adds to the virulence of the general infection of a port, a large number of foreigners in port at such time, causing the disease to spread and rage with extraordinary violence, have repeatedly caused the infection to become so unusually virulent, that many of those who were supposed acclimated, have contracted the disease, and even have died.

As a general rule, the natives of tropical cities seldom experience an attack of the disease; and even where it becomes epidemic among them, as it has been occasionally, it assumes such a mitigated form as to present entirely a different aspect from the same disease in strangers.

Hence the inference is sanctioned by every principle of induction, that Yellow Fever as it presents itself among northern strangers in the West Indies, is an *artificial* disease in those ports, a disease which would be altogether unknown among the natives of the West Indies, were it not for the constant influx of northern strangers into their commercial ports. Even in New Orleans this disease would be almost unknown as an epidemic, were the city protected from the hundreds of strangers and European emigrants, who crowd the wharves and vessels from June to October. The disease could not spread without them; it would be like fire without fuel; it would die by its own action. The infection annually introduced into that city from Havana, and other tropical ports, would be comparatively harmless, were it not for the strangers and unacclimated, who serve as fuel, not only to give action and energy to the fire, but likewise to extend its ravages over the city among those who are imperfectly acclimated. The name of "*Stranger's Fever*," by which it is designated in many tropical ports, indicates its true origin and prevalence.

It is a well established fact, that unacclimated persons, from northern latitudes, who visit an infected port, or city, or who pass through an infected district, or breathe for only a few moments the infected air, will thereby contract Yellow Fever, which will develope itself in five or six days, as a general rule; and often in a less time. The infection is received through the *lungs, by respiration;* and its morbid impression is communicated to the system in the first five minutes of time—and often by the first few respirations. The person who is thus exposed, may travel by steamboat or other conveyance, to remote points, even two or three hundred miles from the point of infection, and there be seized with the disease in its most aggravated form. The distance to which he may travel while the infection is dormant in his system, does not in any wise modify or mitigate the violence of the attack. Thus within a few days, it is possible for twenty or even fifty persons, who have contracted Yellow Fever infection in New Orleans, to be attacked with that disease fatally in Natchez, where all are healthy. This is more likely to occur, as the direct daily intercourse by steamboats between these two points is uninterrupted and extensive. Goods and freight of every kind, and passengers, are continually arriving in great numbers every day; and when the first alarm or declaration, of Yellow Fever as an epidemic in New Orleans, is made public, many strangers and new-comers immediately leave for Natchez and other interior towns. Thus a large supply of infected persons and goods are landed at Natchez within the first three weeks after the disease is epidemic in New Orleans; and a gradual supply is kept up for weeks afterwards. Hence Natchez becomes infected with Yellow Fever within three or four weeks after it has become epidemic in New Orleans. This point and others analogous we shall endeavor to establish as we progress.

The history of every Yellow Fever epidemic in Natchez proves beyond doubt, that those who remain in the city during the gradual increase of the pestilential miasm, acquire a partial immunity to its action, by what is termed acclimation; but when this infectious miasm becomes more virulent from concentration, these partially acclimated persons are generally liable to its attack in its most aggravated form. Natives of New Orleans, and those thoroughly acclimated in cities subject to frequent visitations of Yellow Fever, acquire an entire immunity against its influence. With such there is no apprehension of danger from the disease; and the period of Yellow Fever epidemic is to them the most healthy of the whole summer; for they are at that time liable to no other diseases. This remark we have heard repeatedly from the acclimated citizens of New Orleans, declaring that the period of the epidemic is the most healthy of the whole year, for those who are thoroughly acclimated.

We have said that infected air, such as exists in every part, avenue, recess, and interstice of an infected part of a city, becomes more virulent in proportion to its confined condition, in a temperature but little under that of a tropical summer, or between 85° and 90° of Fahrenheit. If the temperature of the confined air be near or above 80° it will rapidly increase in virulence; and more slowly in proportion as it is below 80°. Confined rooms, ship-holds, and bales and boxes of porous goods, retain the infected atmosphere very near at the usual noon-day temperature; consequently it becomes more virulent than the free portion which is by night reduced 25 or 30° below the noon-day temperature. All porous articles as bales of blankets, and feather beds, contain by far the largest portion of their bulk of air. This air is such as that from which they are removed. A bale of blankets compressed until the whole air is expressed will be reduced to less than one third of its original bulk. A feather-bed of ordinary size contains probably 25 lbs. of feathers, in a bed-tick having a capacity for 24 cubic feet of air. The air constitutes the great bulk of the feather-bed; for if the bed be compressed until the whole air is expelled, the bed will be reduced to a solid form, and to a bulk *not exceeding* two cubic feet at most. Thus we should in an infected feather-bed have about 22 cubic feet of infected air. This can be transported undiluted for any reasonable distance, even 500 miles. The infection carried by such articles in confined vessels becomes more virulent while the temperature is near or above 80°, and constitutes what is known as *fomites*, or the *most virulent infection.* The fomites in such articles becomes much more virulent upon the human system, when, in addition to the free atmospheric infection, from which they have been taken, they have been saturated with the exhalations and secretions, from bodies laboring under Yellow Fever in its most aggravated form. Such *fomites* are active in producing Yellow Fever in those who handle and sleep on those articles. Hence a smaller portion of this grade of infection inhaled, will excite Yellow Fever, than of the free infection. The secretions and exhalations of a body laboring under malignant Yellow Fever, even in a pure air, will so saturate and infect the bed and bedding of the patient, as to be capable of exciting the same disease, and of the same grade, in those who shall weeks afterwards sleep on, or handle them; provided they have been subsequently shut up in a room or confined in a chest. This fact has been repeatedly established in the different visitations of New Orleans and Natchez. Hence it is unsafe for beds, and bedding, to be carried from an infected city into the country and there used: but it is extremely unsafe to carry and use such beds and bedding as were used by patients diseased with Yellow Fever. Cases of this kind will be stated as we progress.

The transportation of a bed or bedding in the confined hold of a ship or steamboat, does not change the nature or virulence of the infected air contained, but it actually becomes more virulent by remaining confined in a close room, under a high temperature. Hence after a trip of three or four days sail under a tropical sun, where the lowest temperature within the vessel is between 88° and 90°, the infected air becomes more virulent than when the vessel was in port, and the danger springing from such source is in the same proportion greater.

An individual, sleeping upon a feather bed, or on other spongy bodies, whether charged with healthy or infected air, necessarily breathes more or less of the air contained. His body presses out of a feather bed, a portion of air equal to the bulk of the body. This air expressed settles around him and is immediately respired; other portions of it are diffused in the contiguous air of the room. Additional portions are successively pressed out by each movement or change of position, and are likewise respired and diffused in the immediate atmosphere. Thus in one night, a healthy individual will press out the greater part of the air contained in the bed on which he sleeps. If that air be infected, he will unconsciously have been breathing more or less of the strongest infection during the night. Should this individual be travelling, he may be attacked with malignant fever several days afterwards, and possibly more than a hundred miles from the place where he received the infection. Such are the circumstances of cases, sometimes adduced by those of contracted observation, to prove that Yellow Fever is sporadic, and of diverse local origins. The disease is seen, and its character is incontestible; but the source from whence it was derived is either forgotten or entirely overlooked.

In this manner, doubtless, are produced many of the cases called sporadic, in the vicinity of infected towns, ships, and houses. Tales of this kind can be passed off currently upon northern men, where genuine Yellow Fever is *scarcely known:* they can also be imposed upon enlightened medical men who have never seen this disease, and who are tied down to received opinions, and pre-conceived theories. To those who live in or near the tropical regions, where Yellow Fever is no stranger, and where all its habits, customs, and characteristics are familiar, these theories pass as an idle dream.

Thus far it matters not from what source, or in what manner, the poison of the Yellow Fever is produced; nor in what manner its effects are produced on the system. We will call it *infection;* and we will admit, for sake of argument, that it is produced from any source which may suit the fancy of the reader. All admit that the local atmosphere of certain places becomes infected with this morbific poison, and that Yellow Fever is the result; that the local atmosphere may be partially, or more strongly charged with this infection, producing the proper Yellow Fever malaria.

As to the nature, composition, and properties of this malaria or poison of Yellow Fever, we know nothing, except from its effects upon the human system. Of its qualities and *modus operandi* upon the human system, we know only by induction: from facts and repeated observation where it prevails, we deduce some of the general laws of its operation.

Its effects upon the healthy system in the production of its peculiar disease, according to Baron Larrey,* is through the medium of the lungs. This view of its operation upon the system is sustained by many distinguished men, who have been familiar with the Yellow Fever in all its grades, in the West Indies as well as in the United States. My friend, Dr. Cartwright of Natchez, very correctly locates its primary action upon the pulmonary tissues, being inhaled with the common air in respiration. He conceives that its morbific influence upon the system is conveyed from the lungs, through the medium of the ganglionic system of nerves; and that the disturbance of all the organs and functions of animal and organic life, consequent, constitutes Yellow Fever.† We consider the point established beyond controversy, that Yellow Fever is communicated chiefly, if not entirely, through the air respired; and not by any contact, or any palpable matter otherwise applied.

One point most unsettled in relation to Yellow Fever is, whether bodies laboring under Yellow Fever are capable of eliminating, or throwing off any exhalation, or effluvium, possessing or deriving peculiar morbific properties. Yet the general clinical regimen, and precautionary measures inculcated, even by those who deny any contagious properties, tend strongly to convince the unprejudiced observer, that *all par-*

---

*See Quarterly Journal of Foreign Med. for April, 1832.

†See Med. Recorder, vol. 9, p. 37–8-9, &c.

*ties do admit* the principle; although some deny the fact. The general admission of those who disclaim prejudice on either hand is, that when Yellow Fever makes its appearance as an epidemic, in any port, city, or town, each and every additional case tends still further to contaminate the air; in other words, to increase the infection which is abroad. Those who have been frequently conversant with this disease in the southern ports and towns of the United States, and in the West Indies, untrammelled by prejudice or theory, and governed by their own repeated observation, admit this fact without hesitation or argument. Those who are altogether unacquainted with the visitations of this disease should hardly hazard a contrary opinion on the subject.

If one body, or any number of bodies, laboring under this disease, in the midst of a dense population, can possibly have any agency or influence in rendering more virulent an atmosphere already infected, it must be by imparting to it some morbific property. It certainly can not proceed from *diminishing* the amount of infection already abroad. Each portion of the malaria consumed by each individual in developing a case of the disease, must certainly diminish the general amount. The multiplication of cases should therefore diminish instead of increasing the virulence of the infection, if some morbific influence were not super-added from each new case. Each new case therefore becomes a new source of atmospheric contamination. If any thing be imparted to the local atmosphere from a case of Yellow Fever, it must be thrown off in gaseous and invisible form from the body; either by respiratory exhalation, or by insensible perspiration from the skin. Observation and experience have fully shown that it is not from any palpable excretion from the alimentary canal, or from any peculiar palpable virus.

The skin and lungs are two of the greatest emunctories of the human system, and carry off in a gaseous form more fluids than all the other excretories together. The skin alone throws off by insensible perspiration, in form of gas or vapor, about five pounds of fluid in twenty-four hours. This in form of vapour will occupy a space in the free air equal to the dimensions of a large room, or equal to the entire size of the largest balloon.*

In the period of twenty-four hours the amount of fluid thrown off from the lungs in a gaseous form, is at least thirty *per cent* greater. Respiration throws off into the air a large amount of contaminating affluvia, and tends to deprive the free air of its healthy properties, even while the body is in a state of perfect health. The quantity of gaseous fluids exhaled from each of these emunctories in disease, such as ardent Yellow Fever cannot be less than in health. Consequently every individual of adult age, laboring under Yellow Fever, throws off into the surrounding atmosphere a volume of vapour equal to at least five thousand cubic feet every twenty-four hours. This is unquestionably morbific to a certain extent, and to that vitiates or contaminates the free air. To what extent then must the atmosphere of a house or vessel be contaminated when there are several cases of malignant Yellow Fever, in a comparatively circumscribed and confined atmosphere? Independently of this contamination, the air is still further vitiated by the change which respiration effects in the properties and constitutional elements of the atmosphere respired.

A portion of the exhalations thrown off by these two great emunctories, in disease, is condensed and absorbed by the bed and bedding used by the patient; the remainder is diffused into the surrounding atmosphere. That portion diffused in the free air becomes dilute, and partially loses its virulence; while that confined in the bed and clothing acquires an increased degree of virulence. If this is not the case why do non-contagionists enjoin strict cleanliness and free ventilation, about Yellow Fever patients?

We are told that those exhalations and secretions are morbific in a general way; but do not produce Yellow Fever especially. Do they fear the production of ordinary disease

*See principles of expansion by evolving latent heat, by Prof. Espy on storms.

more than Yellow Fever from these unhealthy effluvia? If they have any tendency to produce disease at all, it is surely none other than Yellow Fever; the identical disease from which it is eliminated.

We are not prepared to prove at what stage or period of Yellow Fever the morbific effluvia are eliminated, which are most active in producing this disease in healthy individuals. Baron Larrey, with much plausibility, supposes that there is a particular period of Yellow Fever, when the infectious effluvium from the body is more virulent, or is thrown off in greater quantities. He supposes that this period of the disease is of but short duration, after which the effluvia from the body are much less virulent. In this particular, if Yellow Fever presents any analogy to other contagious, or exanthematic fevers, the most contagious or infectious stage must be during the first two days; or while the excitement is active and the febrile *erethem* is upon the surface. After the excitement subsides, and the erethem retires, the activity of the morbific effluvia begins to decline.* The palpable excretions, such as the urine, fæces, and black-vomit discharge are entirely harmless as a cause of Yellow Fever in those who handle or taste them; and equally so when taken into the stomach.

But it is contended by some that the exhalations and excretions of persons laboring under any and every disease, in a confined air, are to a certain extent morbific; and that in this respect, Yellow Fever is at most only such in a higher degree. This is a false argument. The sickly effluvia, and the palpable filth which might accumulate in a close chamber, would certainly offend the senses and produce some disturbance of the functions of organic life. But if this disturbance were uniformly and invariably of the same grade and variety of disease as that from which those offensive excretions emanated, it would be called contagion or infection by the most sceptical theorist.

Does any non-contagionist enjoin the free ventilation and strict cleanliness in Yellow Fever rooms and wards, for fear

*See Townsend on Yellow Fever, of New York, p. 288.

those offensive effluvia may produce in others, remittent fever, rheumatism, or gout, measles, or anthrax? No: they apprehend an aggravation of the Yellow Fever malaria, which is to reproduce in others the *identical disease* of Yellow Fever, and no other. According to their prejudices, this may not be contagion, but it bears a striking analogy. We doubt not that *all epidemics*, to a certain extent, are contagious, or that an accumulation of cases in any one house or street, tends greatly to increase the virulence of the disease in that quarter, as well as its more rapid extension.

There are two kinds of morbific matter eliminated from bodies diseased, and which tend to reproduce the same disease in others. This matter is called contagion or infection. The one is a palpable matter of secretion; the other is an invisible gaseous exhalation. Some diseases throw off one kind of infection and some the other. Others, such as small pox, plague, and malignant erysipelas, throw off both kinds of infection: others again, as syphilis and the vaccine pock, are communicated only by a palpable virus. Of the gaseous infections, some are so mild as scarcely to be estimated, especially in unconfined air. Others are more active, but still are greatly diluted or neutralized by free pure air; such are yellow fever, cholera, typhus gravior, and some others. Some, such as small-pox, throw off such a virulent and subtle effluvium, as to prove infectious under nearly all circumstances, to the distance of a few feet from the patient, and in almost all degrees of temperature. Some diseases disseminate their infectious effluvia in hot sultry air; others in a cool damp atmosphere; others in an atmosphere charged with human exhalations. Each disease, which is not actively contagious or infectious, under all circumstances of seasons and temperature, becomes so under certain peculiar circumstances. Of this class is Yellow Fever, which gives off an effluvium which *becomes* morbific or infectious under the proper circumstances.

In relation to Yellow Fever, the great error of both parties, is *ultraism*. The advocate for contagion or personal infection seems to believe it impossible to originate or disseminate the disease in any other manner. His opponent, equally scrupulous of consistency, believes that certain circumstances independent of personal infection, do sometimes originate Yellow Fever; and therefore the disease, when produced, must be entirely free from any contagious or infectious properties. Upon this last assumption, we may ask, whence did the first case of small-pox originate? Nature is not parsimonious in her operations for the production of disease, any more than in the other exhibitions of her power. A disease may certainly originate from some peculiar exciting circumstances, independent of personal contagion; and still, when once produced, it may possess properties more or less infectious. The first case of contagious disease certainly did not originate from personal contagion, but entirely independent of it. The same causes may still produce similar effects.

If a disease possess the properties of reproduction, even once in a hundred cases, and under the most favorable circumstances, it certainly has some claims to be acknowledged an infectious or contagious disease. The contagion or infection is specific, because in all cases it produces the same disease as that from which the infection emanated. A disease which spreads by personal infection alone, makes but slow advances through any community, and generally from one individual to another in regular and slow succession. Infectious diseases, assuming an epidemic character, proceeding from an infected local atmosphere, attack a number of individuals almost simultaneously, or in close and irregular succession. Yellow Fever proceeds in the latter mode; and each case tends to extend the limit of the first or local source of infection, until checked by frost, or by a strict non-intercourse with the healthy population. Small pox does not spread in a city in this rapid and irregular manner.

Dr. Rush is the great father of the doctrine of the local origin of Yellow Fever, from putrescent matters, and from city filth. The doctrine taught by Dr. Rush on this subject, enforced and promulgated as it was by his popularity, talents, and industry, has doubtless been the destruction of thousands. Had it not been for his influence in the medical community of the United States, our northern sea-ports would not have been so long subject to the pestilential visitations of Yellow Fever. New York, Philadelphia, and Boston, and other ports of less note, would have protected their citizens by a judicious quarantine, at least twenty years sooner than they did. The southern ports, still acknowledging a vassalage to his authority, and to his arbitrary dictation, through his disciples, to this day immolate hundreds and thousands of victims annually upon the altar of a blind incredulity. But the dawn of their emancipation begins to shadow forth a more discriminating era. A short time yet, and Yellow Fever shall be excluded from Charleston, New Orleans, and all the south-western ports of the United States. Then this moloch, set up by Dr. Rush, shall no longer devour its living hecatombs of innocent people for its autumnal repast.

At the time when Dr. Rush sought to establish his theory of a local and domestic origin of Yellow Fever, in contradistinction to that which ascribed it to a foreign source, and to imported infection, people were paralyzed with fear at the horrid ravages made by it in their cities. They believed it communicated *exclusively by personal contagion;* and under this conviction the sick were abandoned to their fate, and deserted by friends, and attendants, to perish alone, in neglected despair. None but the strongest of all human ties, those of parental or conjugal love, were able to keep the friends and attendants where certain death seemed to lay in wait for them. Like men in the plague, the plague-spot was a signal for hopeless despair and solitary death.

Feelings of humanity prompted that good man to combat the belief which seemed to sever all the bonds of friendship, and abandoned the sick to their fate. In his zeal for philanthropy, he plunged into the opposite extreme, and was instrumental in throwing off all means of precaution, and to expose whole cities to be ravaged by the worst of plagues. He exerted every influence and urged every argument to convince the people, that the Yellow Fever, with all its horrors, was free from any contagious or infectious properties; that unacclimated persons, with perfect impunity, might occupy the rooms, beds, and clothes of bodies laboring under this fatal disease, while continually exhaling its pestiferous effluvia. He endeavored to show that the exhalations and excretions from Yellow Fever patients were as free from morbific agency, as those from the simplest intermittent.

If he had stopt here his name might still be blessed by many; but he hesitated not to blind the popular judgment, and induce them to adopt a system of police, calculated in practice, to introduce the pestilence into the ports almost every year. He was indefatigable in his exertions to convince the people that the disease was exclusively of local origin, within their cities, and from causes entirely under their control; that there was no such thing as an infected ship, coming from an infected port; that when infection and death began to spread about the wharves, vessels, and those having intercourse with them, all proceeded from some small source of putrescent matter, to some rotten potatoes, coffee, or some other equally inoffensive substance, which may have been thrown or spilt about the wharves. In this he presumed upon their want of discrimination in such subjects, where he knew the people could not judge. Hence setting aside what appeared to them hypothesis and speculation, he pointed them to such matters as would strike the grosser senses of sight and smell; to city filth in alleys, lanes, and sewers; to putrid animal and vegetable matters;* things too, by the by, which had existed in an equal degree a thousand times before, in the midst of uninterrupted health.

It is now time to arrive nearer the goal of truth; and to point out where truth and error meet; to lay aside the trammels of authority, and the prejudices of education, that we may view things clearly, patiently, and with proper discrimination. To this end we desire to contribute our feeble aid, and hope we shall be heard with patience, and with a desire

*See Rush's Works, vol. 2; edition of 1815; Yellow Fever of 1793; Medical Inquiries.

more to arrive at *truth*, than to sustain any theory or preconceived doctrine.

Notwithstanding the zeal with which Dr. Rush labored to establish his theory of the domestic origin of Yellow Fever in our commercial cities, he has also left on record proof and admissions of all that we contend for, or wish to establish. Although he contends that it is of local origin, and is not contagious, yet he clearly admits that under *certain circumstances* it does reproduce itself; or will spread in *impure* or *miasmatic* air. This air is rendered impure or miasmatic, by certain states of vegetable and animal putrefaction in certain states of the weather. This fact we also admit; but deny any agency to the animal or vegetable putrescency.

Dr. Rush says, "Yellow Fever is not contagious in its simple state, and spreads exclusively by means of exhalations from putrid matters, which are diffused in the air. That it does not spread in the country, when carried thither, from cities in the United States; that it does not spread in Yellow Fever hospitals, where they are situated beyond the influence of the impure air in which it is generated; * * * that it generally requires the co-operation of an *exciting* cause, with miasmata to produce it."*

Yet he admits in another place, that Yellow Fever exhalations and secretions, accumulating in close filthy rooms, even in the country, will become infectious. He says, "I have heard of *two or three instances*, in which Yellow Fever was propagated by these means *in the country*, remote from the place where it originated, as well as from every external source of *putrid exhalation*."† If a disease assume a contagious character only *occasionally*, it should be ranked as a contagious disease.

He also admits that Yellow Fever may be contracted by "a person *sleeping in the sheets, or upon a bed impregnated with the sweats and other excretions*, or by being exposed to the smell (breathing,) of foul linen, or *other* clothing of per-

*See Medical Repository, vol. 6, p. 156, &c.

†Ibid. p. 157.

sons who had the Yellow Fever."* Also, "that it was once once produced in Philadelphia *from the effluvia from* a chest of unwashed clothes, which belonged to one of our citizens who had died with it in Barbadoes; but it extended no further than to the person who opened the chest."

Now what was this if it were not contagion, or infection, created from a patient laboring under disease? a contagion, too, which could remain enclosed in a trunk, in a healthy ship, and in this manner be transported unimpaired in virulence, for 2000 miles? It was a *specific* contagion, producing the *same disease* in a healthy system, as that from which it was eliminated. Dr. Rush also admits that occurrences of this kind would be more frequent, were it not for "the *superstitious dread of contagion*, which has generally produced *great care*, not only in washing sheets and clothes, and airing beds supposed to be infected, but frequently the total destruction of them by fire."† Thus, according to Dr. Rush's admission, Yellow Fever would be imported much more frequently were it not for that "*superstitious dread*" which prompts to every precaution necessary for its exclusion. Strange that he should have labored to prove that this disease is produced exclusively by putrid effluvia about wharves, &c.

At the same time, the united talent and experience of the whole medical profession of the United States and Europe was in direct contradiction to the doctrine of the local origin of Yellow Fever, from filth and putrid effluvia. The College of Physicians, of Philadelphia, in reply to a call from the Governor of the State of Pennsylvania, after mature deliberation, made the following report in 1793, viz:

"No instance has *ever occurred*, of the disease called *Yellow Fever, having been generated* in this city, or in any other parts of the United States, as far as we know; but there have been *frequent instances of its having been imported*, not only into this, but into other parts of North America, and prevailing there for a certain period of time. From the rise, progress, and nature of the malignant fever, which began to prevail here

*Med. Repos. vol. 6, p. 156, &c.

†Ibid.

about the beginning of last August, and extended itself gradually over a great part of the city, we are of opinion that *this disease was imported into Philadelphia* by some of the vessels, which arrived in port after the middle of July. In this opinion we are further confirmed by various accounts received from unquestionable authorities."

Signed by order of the College of Physicians.

John Redman, *President.*

*November* 26, 1793.

This opinion of the College of Physicians was sustained by the great body of the medical faculty in the city, as well as in the United States. Dr. Rush and a few others contended that the whole epidemic of 1793 originated in a lot of spoiled coffee!!* Subsequent epidemics he ascribed to putrid hides and potatoes.

Dr. Litton, an eminent physician and contemporary of Dr. Rush, speaking of the Yellow Fever which prevailed at Wilmington, Delaware, in August, 1798, says: "*No one doubted its having been brought from Philadelphia,* in the infectious bad air in boats and shallops," &c.; also, he says, it began about the water's edge, and spread gradually up to the highest streets; having first appeared in August it became epidemic by the middle of September.†

Relative to the same epidemic, Dr. Geo. Monroe says, it was clearly traced to an infected vessel from Philadelphia, then at the wharf; and that the portion of the town near the wharf suffered most severely.‡ The temperature of the atmosphere, and the state of the season being suitable for the propagation of the disease, it soon assumed an epidemic character. Dr. Rush himself admits, that when the atmosphere is highly infectious, or "charged with the miasmatic effluvia, or 'pestilential exhalations,' *a single case of Yellow Fever will excite* it in a whole family."§

---

*See Med. Inquiries, Yellow Fever of 1793,

†See Med. Repos. vol. 3, p. 128–30.

‡Ibid. p. 136, &c.

§Med. Repos. vol. 6, p. 160.

These facts are not adduced by these men as evidence of *personal contagion* in Yellow Fever; but as showing clearly that the contaminated, or infected air from the holds of ships, may be so diffused about a wharf as to produce Yellow Fever in those who breathe it, after residing in a miasmatic atmosphere. Although they do not admit that there is any peculiar contagion, or infection, contained in such vessels; yet they believe it is an impure, or infectious air, capable of producing Yellow Fever in those who breathe it, and of diffusing itself with morbific virulence in the surrounding air, when the latter is contaminated with miasmatic exhalations; but that in a pure atmosphere, free from these miasmatic exhalations, it is innocent.* This admits a great principle, which is equally important, as if it admitted direct infection or contagion. It admits that from whatever cause, a ship may introduce Yellow Fever from one port into another. Yet they contend, that in a healthy pure atmosphere, this infection will not spread so as to bring on an epidemic; that only those who go on board such vessel are liable to contract disease from it; and that those who are thus attacked can communicate no disease to others unless the air is highly contaminated by miasmatic exhalations.

This contaminated state of the air, which is said to be requisite for the dissemination of Yellow Fever, presents precisely the condition we deem requisite for its propagation as an epidemic. But Dr. Rush and others, compelled to admit the truth and force of these facts, endeavor to evade the true inference, by ascribing all the morbific influence and Yellow Fever predisposition, to a previous state of the local atmosphere; leaving to the malaria from the vessels, only the agency of an *exciting cause.* In this view of the case, the seeds of this peculiar disease had been previously imbibed, and continued in a dormant state, until the *exciting* cause of miasm warmed them into life. This would place the infectious air, or malaria of ships, which is known to produce Yellow Fever, in precisely the same relative agency as fatigue, exposure, or

*Med. Repository, vol. 3, p. 200.

dissipation, in all its forms. On this principle the disease excited into action, should be as various as are the forms of disease induced by such causes.

The followers of Dr. Rush have greatly extended his discoveries into the mysteries of Yellow Fever miasm. Since his time, it has been discovered that, not only putrescent coffee, Irish potatoes, and city filth, which offend the senses, but that numerous other sources are equally active in producing epidemic Yellow Fever. Although all experience and authority concur in admitting that Yellow Fever infection, when prevalent, is an invisible, inodorous, and aerial poison, which cannot be perceived by any of the senses, nor be detected by any chemical analysis, yet the followers of Dr. Rush look exclusively to the *fœtor* of *putrefaction.* When Yellow Fever makes its appearance in a city, or in any port, a search is immediately undertaken for the source of the infectious miasm. The first matters in a state of decomposition, whether animal or vegetable, are at once hailed as the fountain and source of all the pestilential miasm. If neither of these be found in the proper direction, some filth nearly allied to a combination of both are sought and most generally found!!

This continues to be the case, notwithstanding these fœtid effluvia have been tested experimentally, and found to contain no principle actively deleterious to health. Epidemic Yellow Fever is still traced to matters which are offensive to the *olfactories*, notwithstanding all experience proves that the most active infection of Yellow Fever cannot be distinguished by any of the senses from common air. Instead of pursuing the dictates of reason and experience, these inquirers are too frequently "*led by the nose;*" and when nothing else presents of sufficient importance, all the pestilence is traced to a filthy back-yard, a wet sewer, a dead rat, or a rotten Irish potatoe.

Others guided in their observations by a sincere desire to arrive at truth, have been indefatigable in their efforts to discover the true source of this mysterious poison. The medical profession have been unremitting in their exertions to detect the origin and nature of this poison. But all theories which trace its source in the United States, to animal or vegetable putrefaction, or to both jointly, must be abandoned, as inapplicable to our southern ports at least. In many cases acute inquirers have supposed they had traced the monster to his den; but when they have attempted to place the shackles upon him, he has vanished. Others again, repudiating the putrefactive origin, have sought other sources widely different from either. Knowing it to be an invisible gaseous poison, beyond the test of any of the senses, they have directed their inquiries to atmospheric causes. At one time it has been traced to a long continuance of high temperature at certain seasons of the year. At another time it has been traced to great and sudden vicissitudes of weather; such as heat and cold. At other times, to electricity, or to a mysterious epidemic constitution of the atmosphere, without any other definite idea. All these, at times, may have exerted some influence or agency, in modifying the action of the poisonous miasm; but they certainly could not have been the primary active source of the disease. They may have increased or diminished the susceptibility of the system to its action, or so modified that susceptibility, as to cause the morbific effects upon the system, to appear more or less the immediate consequence, from the efficient malaria, or infection.

In the further consideration of this subject, we shall briefly examine the most prominent theories or hypotheses relative to the prime origin of the Yellow Fever epidemic miasm. We shall next show, from American authorities, that Yellow Fever has been repeatedly introduced by infected vessels into the ports of the United States, and that the most malignant epidemics were the consequence. Our views of the manner of introduction and dissemination in the south-western ports, will be fully exemplified in treating of the epidemics of Natchez, as well as in relation to the prevalence of Yellow Fever in many ports and towns of the south-west in the summer and autumn of 1839.

(TO BE CONTINUED.)

